# Physical properties and hydration behavior of a fast-setting bioceramic endodontic material

**DOI:** 10.1186/s12903-016-0184-1

**Published:** 2016-02-20

**Authors:** Ya-juan Guo, Tian-feng Du, Hong-bo Li, Ya Shen, Christophe Mobuchon, Ahmed Hieawy, Zhe-jun Wang, Yan Yang, Jingzhi Ma, Markus Haapasalo

**Affiliations:** Institute of Stomatology, Chinese PLA General Hospital, Beijing, China; Department of Stomatology, the First Affiliated Hospital of Zhengzhou University, Zhengzhou, China; Division of Endodontics, Department of Oral Biological & Medical Sciences, Faculty of Dentistry, The University of British Columbia, 2199 Wesbrook Mall, Vancouver, BC V6T 1Z3 Canada; Department of Materials Engineering, The University of British Columbia, Vancouver, Canada; Department of Stomatology, Tongji Hospital, Tongji Medical College, Huazhong University of Science and Technology, Wuhan, China

**Keywords:** Calcium phosphate silicate cement, Calcium silicate-based cement, Differential scanning calorimetry, Microhardness, Mineral trioxide aggregate, Physical properties, Setting reaction

## Abstract

**Background:**

To investigate the physical properties and the hydration behaviour of the fast-setting bioceramic iRoot FS Fast Set Root Repair Material (iRoot FS) and three other endodontic cements.

**Methods:**

iRoot FS, Endosequence Root Repair Material Putty (ERRM Putty), gray and white mineral trioxide aggregate (G-MTA & W-MTA), and intermediate restorative material (IRM) were evaluated. The setting time was measured using ANSI/ADA standards. Microhardness was evaluated using the Vickers indentation test. Compressive strength and porosity were investigated at 7 and 28 days. Differential scanning calorimetry (DSC) was employed for the hydration test.

**Results:**

iRoot FS had the shortest setting time of the four bioceramic cements (*p* < .001). The microhardness values of iRoot FS, ERRM Putty and MTA increased at different rates over the 28 days period. At day one, ERRM Putty had the lowest microhardness of the bioceramic cements (*p* < .001), but reached the same level as MTA at 4, 7 and 28 days. The microhardness of iRoot FS was lower than that of W-MTA at 7 and 28 days (*p* < .05). The porosity of the materials did not change after 7 days (*p* < .05). The compressive strength values at 28 days were significantly greater for all bioceramic groups compared to those at 7 days (*p* < .01). ERRM Putty had the highest compressive strength and the lowest porosity of the evaluated bioceramic cements (*p* < .05), followed by iRoot FS, W-MTA, and G-MTA, respectively. DSC showed that iRoot FS hydrated fastest, inducing an intense exothermic reaction. The ERRM Putty did not demonstrate a clear exothermic peak during the isothermal calorimetry test.

**Conclusions:**

iRoot FS had a faster setting time and hydrating process than the other bioceramic cements tested. The mechanical properties of iRoot FS, G-MTA and W-MTA were relatively similar.

## Background

The first hydraulic calcium silicate-based cement (HCSC) patented for endodontic applications was mineral trioxide aggregate (MTA; Dentsply Tulsa Dental Specialties, Johnson City, TN, USA) [1]. It has attracted considerable attention [2–4] owing to its excellent sealing ability, biocompatibility, regenerative capabilities, and antibacterial properties [2, 3, 5–7]. The main hydraulic components in HCSCs are tricalcium silicate (Ca_3_SiO_5_ or C_3_S) and dicalcium silicate (Ca_2_SiO_4_ or C_2_S). HCSCs have been widely used as both endodontic repair materials and dentin substitutes [8]. An increasing number of publications report that these cements produce an apatite-rich surface layer after they contact simulated body fluids [4, 5, 9]. Several HCSC based root repair materials have been developed following the introduction of MTA and are available clinically for dentists. These include ProRoot (Dentsply Tulsa Dental Specialties), MTA Plus (Prevest-Denpro, Jammu City, India), and BioAggregate (Innovative Bioceramix, Vancouver, Canada). However, there are some drawbacks associated with the use of HCSCs including long setting times, difficulty with manipulation, limited resistance to washout before setting, and the possibility of staining the tooth structure [3, 4, 10]. Therefore, new root repair materials are continually being developed to further improve their properties.

Calcium phosphate silicate cement (CPSC) is a new generation biological cement first proposed in 2006 [11]. It consists of phosphate salts in addition to hydraulic calcium silicates. The reason for its development was the expectation that the hydration process would enhance the cement’s mechanical properties and biocompatibility [12]. As examples of CPSCs [13], Endosequence Root Repair Material Putty (ERRM Putty; Brasseler USA, Savannah, GA, USA) and Endosequence Root Repair Material Paste (ERRM Paste; Brasseler, USA) have been developed as ready-to-use, premixed bioceramic materials. Their major inorganic components include C_3_S, C_2_S, and calcium phosphates. The introduction of premixed CPSCs eliminates the potential of heterogeneous consistency during on-site mixing. Because the material is premixed with nonaqueous but water-miscible carriers, it will not set during storage and hardens only on exposure to an aqueous environment [14]. Both ERRM Putty and Paste have reasonably good handling properties; their working time is more than 30 min and their setting time is 4 h [15]. However, the long setting time is one of the potential drawbacks of HCSCs and CPSCs, consequently two appointments are required with a related increase in chair-side time.

Recently, a CPSC iRoot FS Fast Set Root Repair Material ([iRoot FS]; Innovative Bioceramix) has been introduced for use as a root canal repair material, as a fast setting white hydraulic premixed bioceramic paste (http://www.ibioceramix.com/products.html). iRoot FS is an insoluble, radiopaque and aluminum-free material based on calcium silicate, which requires the presence of water to set and harden. A quickly setting cement could allow for a reduction in chair-side time and the number of visits needed per treatment. However, the fundamental properties of this improved performance material are still unknown. Differential scanning calorimetry (DSC) is a thermal analysis technique well suited to the study of chemical reactions and phase transformations in a wide range of materials. DSC can be used to study the setting of cements by measuring the temperature (i.e., the exothermic heat) during the early stages of setting, as well as monitoring the reaction products that form via their decomposition upon heating [16, 17]. The study of the kinetics of the setting reaction could provide significant information on new materials. Therefore, the purpose of this study was 1) to evaluate the physical properties of iRoot FS, including the setting time, microhardness, compressive strength and porosity, and compare these with ERRM Putty and gray and white ProRoot MTA (G-MTA & W-MTA; Dentsply Tulsa Dental Specialties) as well as an intermediate restorative material (IRM; Dentsply Caulk, Milford, DE, USA); and 2) to investigate the hydration behavior of the cements using DSC analysis.

## Methods

Two commercially available HCSC, G-MTA (batch 12120401B) and W-MTA (batch 11004159) were used in the present study as well as two CPSC-based cements, ERRM Putty (batch 1306 BPP) and iRoot FS (batch 1201FSP-T). IRM was included as a control material (Dentsply Caulk; batch 091214).

### Setting time

The MTA and IRM were mixed and manipulated in accordance with the manufacturer’s instructions. Molds with an inner diameter of 10 mm and a height of 2 mm were used for the MTA and IRM. The molds were placed on a glass plate and the mixed materials were packed into them. The whole assembly was then transferred to an incubator (37 °C, > 95 % relative humidity). For the iRoot FS and ERRM Putty, which require continuous exposure to moisture during setting [18], plaster of Paris molds with a cavity of 10 mm diameter and 2 mm height were used. The molds were first stored at 37 °C in a water bath for 24 h, and then the iRoot FS and ERRM Putty were poured into these molds. The whole assembly was then stored in a water bath at 37 °C.

The initial and final setting times of all samples were in accordance with the American Society for Testing and Materials (ASTM) International Standard C266-03 [19] and the American National Standards Institute/American Dental Association (ANSI/ADA) Specification No. 57 [20]. The Gilmore needle for testing the initial setting time had a weight of 100 g and an active tip of 2.0 mm diameter (initial needle). The needle for the final setting time had a weight of 400 g and an active tip of 1.0 mm diameter (second needle) [21]. The initial needle was applied lightly on the surface of each sample. This procedure was repeated every 5 min for all bioceramic cements and every 2 min for IRM until the needle did not create a complete circular depression on the specimen surface. For each sample, the time that elapsed between the end of mixing and the unsuccessful indentation was recorded in minutes and defined as “the initial setting time”. “The final setting time” was determined following the same procedures using the second needle, with the 400 g load. Five parallel sets of measurements were made for each material.

### Microhardness testing

Microhardness of the set of cements was evaluated using the Vickers indentation test (MICROMET 3, Buehler Ltd., Lake Bluff, IL, USA). Each specimen was tested at 1, 4, 7 and 28 days, at three points with 3 mm intervals and a load of 100 g for 10 s. According to the pilot study, this load created a clear and reliable indent in all materials. Five samples of each material in each group were prepared. The tests were performed on surfaces polished with 1200 grit sand paper using a diamond indenter; the indentation size (i.e. diagonal *d*) was measured and converted to a hardness value as HV [kg/mm^2^] = 0.0018544 *L/d* [22].

### Compressive strength

The sample sizes for compressive strength were 6 mm in diameter by 12 mm in height. The compressive strength of specimens was determined according to the method recommended by ANSI/ADA No. 96 [23] using a universal testing machine (Instron 3369, Instron Co., Norwood, MA, USA). The crosshead speed was 1 mm/min along the long axis. The compressive strength σc [MPa] was calculated using the following Eq. 1). The specimens were kept in 37 °C distilled water for pre-set periods of 7 and 28 days, respectively. At least five specimens were used for each determination.

1 $$ {\upsigma}_{\mathrm{c}}=4\mathrm{P}/\pi {\mathrm{D}}^2 $$

where *P* is the maximum load, N; *D* is the mean diameter of the specimen, mm.

### Porosity

The specimens were kept in 37 °C distilled water for pre-set periods of 7 and 28 days. The porosity was determined using the test method described in ASTM Standard C830-00 [24]. Kerosene was chosen as the saturation liquid instead of water to avoid any reaction with the specimen [24]. The air-dried specimens were dried in an oven at 105 °C to a constant weight and the dry weight, *B*, was determined (for all weight measurements, the gram was the unit used with an accuracy of 0.001 g). The test specimens were then placed in a beaker containing kerosene and located in a vacuum chamber with an absolute pressure of not more than 6.4 kPa for 60 min. At least five measurements were taken for each group. The suspended weight, *S*, was determined for each test specimen suspended in kerosene. The saturated weight, *W*, was determined by removing all drops of liquid from the surface using a wet smooth linen. The exterior volume was calculated by Eq. 2), the volume of open pores was calculated by Eq. 3), and the apparent porosity of the specimen was calculated by Eq. 4).

2 $$ {V}_1 = \left(W\hbox{--}\ S\right)\ /\ \gamma $$

3 $$ {V}_2 = \left(W\hbox{--}\ D\right)\ /\ \gamma $$

4 $$ P = \left({V}_2/{V}_1\right)\times 100\ \% $$

where *V*_*1*_ is the exterior volume of the specimen, cm^3^; *W* is the saturated weight, g; *S* is the suspended weight, g; *γ* is the density of kerosene, 0.80 g/cm^3^; *V*_*2*_ is the volume of open pores, cm^3^; *P* is the apparent porosity, %; *D* is the dry weight, g.

### Differential scanning calorimetry

The kinetics of the setting reactions of the all samples was evaluated with an isothermal calorimeter (DSC Q2000, TA Instruments, New Castle, DE, USA) at a constant temperature of 37 °C [25]. The samples were mixed and manipulated in accordance with the manufacturer’s instructions. The mixtures were transferred to pre-weighed 40-mL aluminum crucibles and weighed in an analytical balance so the amount of mixture in each could be calculated. The ERRM Putty and iRoot FS were mixed with 10 % distilled water (v/v) because they need to absorb moisture to initiate the setting reaction. The sample preparation process was completed in 1 min. The heat flux was automatically recorded every 2 s. Each crucible was fitted with a lid to prevent water evaporation and placed in the DSC for 6 h to analyze any exothermic peaks associated with the setting reactions. As a reference, an empty 40-mL aluminum crucible was used. All resulting DSC thermograms were evaluated by the DSC manufacturer’s software (TA Instruments). Individual specimens were only tested once. Each cement was tested twice.

The results were analyzed using one-way ANOVA or two-way ANOVA with post hoc analysis using software (SPSS for Windows 11.0, SPSS, Chicago, IL, USA) when necessary at a significance level of *p* < 0.05.

## Results

IRM had the shortest initial and final setting time of all tested cements. In the four bioceramic groups, iRoot FS had the shortest initial and final setting time of the CPSCs and HCSCs (*p* < .001) (Table 1). The initial and final setting time of ERRM Putty was longer than W-MTA (*p* < .001). There was no significant difference in the initial and final setting time between ERRM Putty and G-MTA.

**Table 1:** The initial and final setting time (min) of the five materials measured

	G-MTA^c,d^	W-MTA^e^	ERRM Putty^d^	iRoot FS^f^	IRM^g^
Initial setting time (min)^a^	58.3 ± 2.2	42.2 ± 2.1	61.8 ± 2.5	18.3 ± 2.6	7.2 ± 1.1
Final setting time (min)^b^	217.2 ± 17.3	139.6 ± 10.3	208.0 ± 10.0	57.0 ± 2.7	10.8 ± 1.1

Different superscript letters indicate statistically significant differences between groups (*p* < .05)

**Fig 1:**
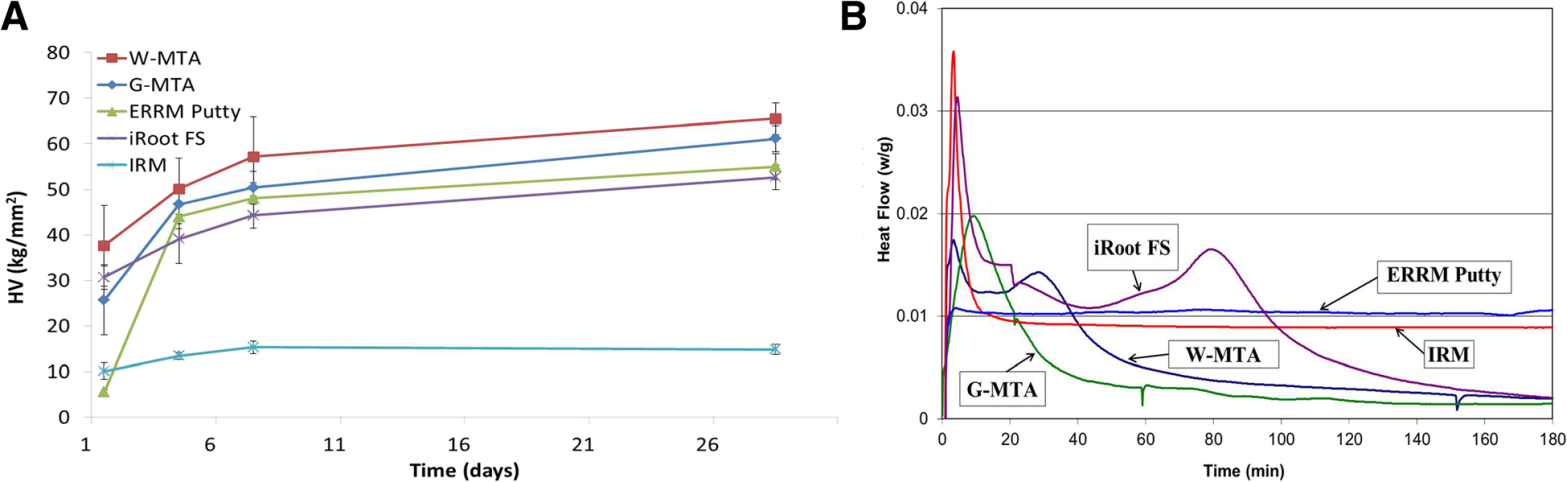
**a** Microhardness values [kg/mm^2^] of MTA, ERRM Putty, iRoot FS and IRM at 1, 4, 7 and 28 days after mixing. **b** Graphical representation of the heat flux generated with time for the different materials

The microhardness of all materials gradually increased over the 28 days period (Fig. 1a). At one-day of setting, ERRM Putty had the lowest microhardness among the four bioceramic cements (*p* < .001), but reached the same level as MTA at 4, 7 and 28 days. There was no significant difference amongst G-MTA, W-MTA, ERRM Putty and iRoot FS at 7 and 28 days. The microhardness of iRoot FS was lower than W-MTA at 7 and 28 days (*p* < .05). IRM had had the lowest microhardness of all tested cements at 28 days.

The compressive strength values at 28 days were significantly greater for all bioceramic groups compared to those at 7 days (*p* < .01) (Table 2). IRM had the lowest compressive strength of all tested materials at 7 and 28 days. There was no significant difference in porosity of the experimental groups between 7 and 28 days. ERRM Putty had the highest compressive strength and lowest porosity (*p* < .05) of the CPSCs and HCSCs.

**Table 2:** Compressive strength (MPa) and porosity (%) of G-MTA, W-MTA, ERRM Putty, iRoot FS and IRM after 7 & 28 days

	Compressive strength (MPa) (((MPa)		Porosity (%)	
	7 days^a^	28 days^b^	7 days	28 days
G-MTA	47.8 ± 12.3^c^	73.6 ± 14.1^d^	28.9 ± 2.2^h^	27.1 ± 1.1^h^
W-MTA	49.6 ± 12.4^c,g^	78.3 ± 16.0^d^	31.4 ± 2.3^h^	30.0 ± 1.6^h^
ERRM Putty	107.4 ± 31.1^e^	176.6 ± 22.0^f^	16.7 ± 2.8^i^	14.3 ± 1.1^i^
iRoot FS	56.6 ± 5.9^g^	96.0 ± 24.3^e^	20.8 ± 2.7^j^	21.6 ± 2.2^j^
IRM	40.6 ± 6.4^c^	49.1 ± 8.0^c,g^	12.9 ± 2.4^i^	12.0 ± 2.3^i^

Different superscript letters indicate statistically significant differences between the materials in different groups (*p* < .05)

The results of DSC isothermal calorimetry are illustrated in Fig. 1b. W-MTA showed two exothermic peaks, a small and narrow peak (0.017 W/g) between 2 to 16 min, and a broad peak between 18–60 min. G-MTA had one strong exothermic peak (0.019 W/g) between 4–50 min. iRoot FS showed two exothermic peaks: a strong and narrow peak (0.031 W/g) between 2–15 min and a broad large peak between 40–100 min. The ERRM Putty did not show a clear exothermic peak during the isothermal calorimetry test. The rate of heat flux of IRM presented a strong (0.036 W/g) and narrow exothermic peak starting at 2 min and ending at 16 min, indicating the time and duration of setting reactions of IRM.

## Discussion

An important factor in non-surgical as well as surgical restorative repair in endodontics is to achieve a fluid-tight seal between the tooth and the repair material [26, 27]. In most cases a bioceramic material is the restorative material of choice. The main disadvantage of currently available bioceramic materials is a setting time of approximately 3 to 4 h [2, 3, 28], which compromises the application, especially in supracrestal areas. The possibility of the material being washed out at cervical/furcal area during the long setting time needs to be considered [27]. In addition, early occlusal pressure directed to the material, even in a deeper location, may compromise the integrity of the seal [27]. Therefore, a bioceramic material that has optimal mechanical behavior and sets fast, would be attractive to the clinician in specific clinical situations. G-MTA and W-MTA were chosen in the present study as gold standard materials because they are widely used for retrograde filling, apexification and perforation repair in endodontic treatment. Although the details of the reaction mechanisms of the new CPSCs remain unknown, the results of the present study showed that iRoot FS had the shortest setting time of the CPSCs and HCSCs. The shortest setting time of iRoot FS may benefit some clinical challenge cases with time demanding. However, clinical study is still required to evaluate its performance.

Most of the hydration of these cements occurs during the first several days, although complete hydration may even take one or two years [4, 9]. The point of maximum exothermic heat generation has been used as an indication of the setting time of various dental cements [16, 17]. Two exothermic peaks were found in the iRoot FS and W-MTA. The first peak possibly correlated with the initial water absorption on the calcium silicate particles surface, followed by their dissolution and the start of hydration of the calcium silicates in the cements. The second peak can be related to the start of calcium hydroxide precipitation, mostly on the surface, which is a by-product of calcium silicate hydration [16]. An early strong peak of iRoot FS was in accordance with our setting time results: iRoot FS had the shortest setting time among the CPSCs and HCSCs. It showed that the isothermal DSC analysis can provide a more complete understanding of the setting property of the cements. Interestingly, while G-MTA had one intense exothermic peak, W-MTA had two peaks. The hydration mechanism of G-MTA is expected to be the same as W-MTA, but the chemical components and particle size distribution could be different, thus affecting the hydration kinetics. No clear exothermic peak was found on ERRM Putty. Therefore, a more advance technique may be required to accurately evaluate the hydration process of ERRM Putty in-depth.

The surface microhardness of a material provides some indication of the surface strength of the material [29]. In the present study, the microhardness values of all cements gradually increased over the 28-day period, which was demonstrated by an early study with G-MTA and W-MTA [30]. Interestingly, the rate of hardening of ERRM Putty was very low during the first day. However, the microhardness of ERRM Putty increased thereafter and reached the same level as the other bioceramic cements at day four. The results showed that all bioceramic cements used in the present study need at least 7 days for complete setting.

Compressive strength is one of the indicators of the setting and strength of a material. Failure in compression is complex, because both the mode and plane of failure are variable. Failure can occur by plastic yielding, cone failure, or by axial splitting [31]. In principle, the mode of failure depends on the size and geometry of the specimen, as well as the precise nature of the material being tested and the rate of loading [31]. This test measures the material’s ability to withstand compression. Higher strength is more desirable, although no clinically relevant minimum, e.g. in endodontics, has been universally proposed. Walsh et al. [32] evaluated the compressive strength of ERRM Putty after exposure to saline and fetal bovine serum. The results showed that the compressive strength value was 40–45 MPa at 7 days, which was lower than the present study. The possible reasons for this variation between the two studies (the present study and Walsh et al. [32]) may be different methodologies in the incubation environment and different dimensions of prepared samples (5 × 4.17 mm *vs* 12 × 6 mm in the present study). In the present study, ERRM Putty had the highest compressive strength among the cements. This may be attributed to the slow hydration process and small size of porosity of ERRM Putty. Porosity has a significant role in the relationship between mechanical properties of calcium silicate cements, such as the compressive strength-modulus of elasticity relationship [33]. Indeed, ERRM Putty had the lowest porosity among the CPSCs and HCSCs in the present study. Torabinejad et al. [34] reported that the compressive strength of G-MTA after 24 h was 40 MPa, and it increased to 67 MPa after 21 days. Their findings lend support to our results: the compressive strength for all bioceramic cements increased with time. The present results revealed that the compressive strength of iRoot FS, G-MTA and W-MTA were relatively similar and stable mechanical properties of bioceramic cements can be obtained after 1 month.

## Conclusions

In conclusion, iRoot FS had a faster setting time and hydrating process than the other bioceramic cements tested. The mechanical characteristics of iRoot FS, G-MTA and W-MTA showed no major differences; HCSC cements (MTAs) had a slightly higher final hardness than the CPCSc cements, while the opposite was true regarding the compressive strength.
